# A Dynamic Impact Simulation Method for Titanium-Based Functionally Graded Composites Based on Abaqus/Explicit: Parametric Analysis, Mesh Strategy, Numerical Artifact Mitigation, and CAE Modeling

**DOI:** 10.3390/ma19163505

**Published:** 2026-08-19

**Authors:** Xinghai Shao, Wenyan Wang, Jingpei Xie, Bobo Li, Zhiping Mao

**Affiliations:** 1 College of Materials Science and Engineering, Henan University of Science and Technology, Luoyang 471023, China; shaoxh@lamic.com.cn (X.S.); zpmao@haust.edu.cn (Z.M.); 2 Qingyan (Luoyang) Technology Industry Co., Ltd., Luoyang 471003, China; 3 State Key Laboratory of Light Superalloys, Henan University of Science and Technology, Luoyang 471003, China; 4 Luoyang Shuangrui Precision Casting Titanium Industry Co., Ltd., Luoyang 471003, China; libobo0816@126.com

**Keywords:** titanium matrix composite, TC4 titanium alloy, functionally graded material, abaqus/explicit, dynamic ballistic impact

## Abstract

Homogeneous TC4 titanium alloy suffers from the strength–ductility trade-off and exhibits insufficient anti-penetration capacity under high-strain-rate impact. TiC_p_-reinforced functionally graded titanium matrix composites (FGTMCs) with a “hard outer, tough inner” gradient architecture are promising lightweight armor materials. The use of Abaqus/Explicit finite element simulation for FGTMCs under dynamic impact can capture transient deformation and damage evolution while enabling rapid evaluation of the impact resistance of different materials; however, research in this area remains scarce. This study conducts a systematic parametric analysis of key simulation parameters, including calibration of the Johnson–Cook constitutive and damage model parameters for TC4 titanium alloy, optimization of mesh partitioning strategies, hourglass control schemes, model dimensions, and boundary conditions to suppress “ghost mesh” numerical artifacts. Material property assignments for TC4 and three typical titanium matrix composites are designed, along with a methodology for constructing functionally graded material models. Two projectile–target matching configurations (small projectile/thin target vs. large projectile/thick target) are compared, and the optimal model of a 700 m/s small-caliber tungsten projectile impacting a 50 mm TC4 target is identified. Parametric analysis demonstrates that a damage parameter D4 = 0.1 significantly improves numerical stability, and a graded mesh strategy with further refinement along the penetration path balances computational accuracy and efficiency. Using the optimized material system, the simulation results reproduce the three-stage damage evolution of titanium alloys under impact—cratering, plastic penetration, and back-face spallation—providing reliable numerical support for the structural optimization of graded armor materials.

## 1. Introduction

Titanium alloys, particularly Ti-6Al-4V (TC4), have attracted considerable research interest in protective armor applications owing to their exceptional combination of low density, high specific strength, excellent corrosion resistance, and good ballistic performance [1,2,3,4,5]. Compared with traditional armor steels and aluminum alloys, titanium alloys offer weight savings of 35–41% while maintaining comparable or superior ballistic protection [6,7,8]. The impact resistance of titanium alloys depends critically on their microstructure—grain size, phase morphology, and texture [9,10,11]. Refining α/β lamellae or equiaxed grains below 10 μm enhances strength and hardness via Hall–Petch strengthening, improving penetration resistance. Yet excessive refinement sacrifices ductility and fracture toughness, promoting adiabatic shear banding and premature failure under dynamic loading. The β-phase cooling rate and post-heat treatments govern α-phase morphology and β-stabilizer distribution, thus modulating strain hardening and dynamic recrystallization. Hence, an optimal microstructure must balance grain refinement with adequate toughness to maximize ballistic performance. The incorporation of ceramic reinforcing phases such as TiC and TiB into titanium matrix composites (TMCs) further enhances the hardness and strength of these materials. Among them, TiC_p_-reinforced functionally graded titanium matrix composites (FGTMCs) with a “hard outer, tough inner” gradient architecture are particularly promising lightweight armor materials [12,13,14,15].

However, the development and optimization of titanium-based armor materials face significant challenges. Experimental ballistic testing is expensive, time-consuming, and provides limited insight into the complex, transient deformation and failure mechanisms that occur during projectile penetration [16,17]. The dynamic impact process involves extremely high strain rates (10^3^–10^5^ s^−1^), severe plastic deformation, adiabatic heating, and complex stress wave propagation—phenomena that are difficult to fully capture through experimental observation alone [18]. Consequently, numerical simulation has become an essential complement to experimental testing in the design and optimization of armor materials.

The Abaqus/Explicit finite element code is widely recognized for its capability to simulate high-speed, short-duration impact events [19,20,21,22]. Its explicit dynamic analysis framework is particularly well-suited for problems involving complex contact conditions, large deformations, and material failure—all of which are characteristic of ballistic impact [23]. The Johnson–Cook (J-C) constitutive model and its associated damage criterion have been extensively employed to describe the rate-dependent, temperature-dependent plastic flow and fracture behavior of metallic materials under impact loading [24,25]. For TC4 titanium alloy, numerous studies have reported J-C model parameters calibrated through experiments at various strain rates and temperatures [26,27,28]. The use of Abaqus/Explicit finite element simulation for FGTMCs under dynamic impact enables rapid evaluation of the impact resistance of different materials; however, research in this area remains scarce.

Despite the widespread use of Abaqus/Explicit in impact simulations, several critical issues remain inadequately addressed in the literature. The selection of appropriate mesh density, hourglass control strategies, and analysis step settings significantly influences both simulation accuracy and computational efficiency. In particular, the emergence of hourglass elements—also referred to as “ghost mesh” or zero-energy deformation modes—in the central deformation region of impacted targets is a persistent numerical artifact that can severely compromise simulation reliability. These hourglass elements exhibit unrealistic deformation patterns without associated strain energy, leading to erroneous stress and strain predictions. While various hourglass control formulations are available in Abaqus/Explicit, their effectiveness in high-strain-rate impact simulations of titanium alloys has not been systematically evaluated.

Furthermore, the selection of appropriate projectile–target configurations for simulation studies remains a practical challenge. Large-mass projectile/thick-target models, while more representative of actual armor penetration scenarios, demand substantial computational resources and are particularly susceptible to hourglass instabilities. Small-caliber projectile/thin-target configurations may offer improved numerical stability and computational efficiency, but their suitability for capturing the essential physics of penetration needs to be carefully assessed.

This study aims to address these gaps by presenting a systematic investigation of dynamic impact simulation methods for TC4 titanium alloy and its composites using Abaqus/Explicit (Abaqus/CAE 2016). The specific objectives are: (1) to establish a reliable finite element modeling framework incorporating the Johnson–Cook constitutive model and damage criterion, and to design material property assignments for TC4 and three typical titanium matrix composites along with a methodology for constructing functionally graded material models; (2) to conduct a parametric analysis of key simulation parameters including mesh density, hourglass control, and analysis step settings; (3) to identify and mitigate the hourglass element issue in high-strain-rate impact simulations; and (4) to compare different projectile–target configurations and recommend optimal simulation strategies. The findings are expected to provide practical guidelines for researchers and engineers engaged in the numerical simulation of ballistic impact on titanium-based armor materials.

## 2. Materials and Methods

### 2.1. Material Model

#### 2.1.1. Johnson–Cook Constitutive Model

The mechanical behavior of TC4 titanium alloy under high-strain-rate impact is described using the Johnson–Cook (J-C) constitutive model [24,25]. This phenomenological model accounts for strain hardening, strain-rate hardening, and thermal softening effects through a multiplicative formulation:

(1) $$\sigma =(A+B\varepsilon^{n})(1+Cln{\dot{\varepsilon }}^{*})(1-T^{*m})$$

where

σ—Mises equivalent flow stress;

ε—Equivalent plastic strain;

${\dot{\varepsilon }}^{*}=\dot{\varepsilon }/{\dot{\varepsilon }}_{0}$—Dimensionless plastic strain rate, with $\dot{\varepsilon }$ as the current plastic strain rate and ${\dot{\varepsilon }}_{0}$ the reference strain rate (usually taken as 10^−3^ or 10^−4^ s^−1^);

T* = (T – T_r_)/(T_m_ – T_r_)—Homologous temperature, reference temperature Tr = 293 K, melting point Tm = 1933 K;

A—Initial yield strength at the reference strain rate and reference temperature (Mpa);

B—Strain hardening coefficient (Mpa);

*n*—Strain hardening exponent (dimensionless);

C—Strain rate sensitivity coefficient (dimensionless);

m—Thermal softening exponent (dimensionless).

The J-C fracture criterion is used to define the strain at which damage initiation occurs:

(2) $$\varepsilon_{f}=\left[D_{1}+D_{2}exp\left(D_{3}\sigma^{*}\right)\right]\left(1+D_{4}\ln{\dot{\varepsilon }}^{*}\right)\left(1+D_{5}T^{*}\right)$$

where

$\sigma^{*}=\sigma_{m}/\bar{\sigma }$—the stress triaxiality;

$\sigma_{m}$—The mean stress;

$\bar{\sigma }$—The von Mises equivalent stress;

D_1_–D_5_—Damage parameters.

#### 2.1.2. Preliminary Material Property Assignment for TC4

To construct a simulation model for TC4-based functionally graded titanium matrix composites (FGTMCs), it is first necessary to define the material properties of the TC4 matrix. Subsequently, the material properties of the composite layers are determined based on experimental experience and design requirements, before finally constructing the FGTMC model. The preliminary J-C constitutive parameters adopted in this study are based on published experimental data and are summarized in Table 1 [26,27,28,29]. Parameter Sets 1 to 3 are widely adopted TC4 material constants reported in existing literature. For a more comparative analysis of Johnson–Cook (J-C) parameters, the following uniform adjustments have been applied to Sets 1–3:

(1) All constants except the damage coefficients D1–D5 are unified to identical values across the three datasets.
(2) The original D1 value of 0.09 in Parameter Sets 1 and 2 is revised to 0.1 to match the D1 magnitude used in Parameter Set 3.
(3) It should be emphasized that the five damage constants D_1_–D_5_ in the J-C fracture criterion only control the critical strain of damage initiation. After damage onset, a displacement-based damage evolution law is utilized in this research rather than an energy-based evolution formulation. The uniform damage evolution displacement of all material systems listed in Table 1 is set to 0.01 mm.

### 2.2. Finite Element Model

#### 2.2.1. Geometry and Assembly

Table 2 summarizes the impact velocities encountered in various protective scenarios. It can be observed that the projectile impact velocities differ significantly across different loading conditions. The protection object and projectile type directly determine the impact velocity regime and the corresponding physical mechanisms. Armor-piercing projectiles and shaped charge jets are primarily used in high-velocity penetration scenarios for armor protection. Shaped charge jets can reach impact velocities up to 3000 m/s, relying on high-velocity jets to achieve efficient penetration of metallic or reactive armors, with complex physical mechanisms governing the impact process. Split Hopkinson Pressure Bar (SHPB) dynamic mechanical testing, on the other hand, represents a medium-to-low strain-rate regime with impact velocities of only 4–35 m/s, commonly employed to obtain dynamic mechanical properties of metallic materials such as titanium alloys. Flat-nosed projectiles, with impact velocities ranging from 160 to 320 m/s, cover a broad range of applications and are frequently used as a loading method in impact simulations.

**Figure 1:**
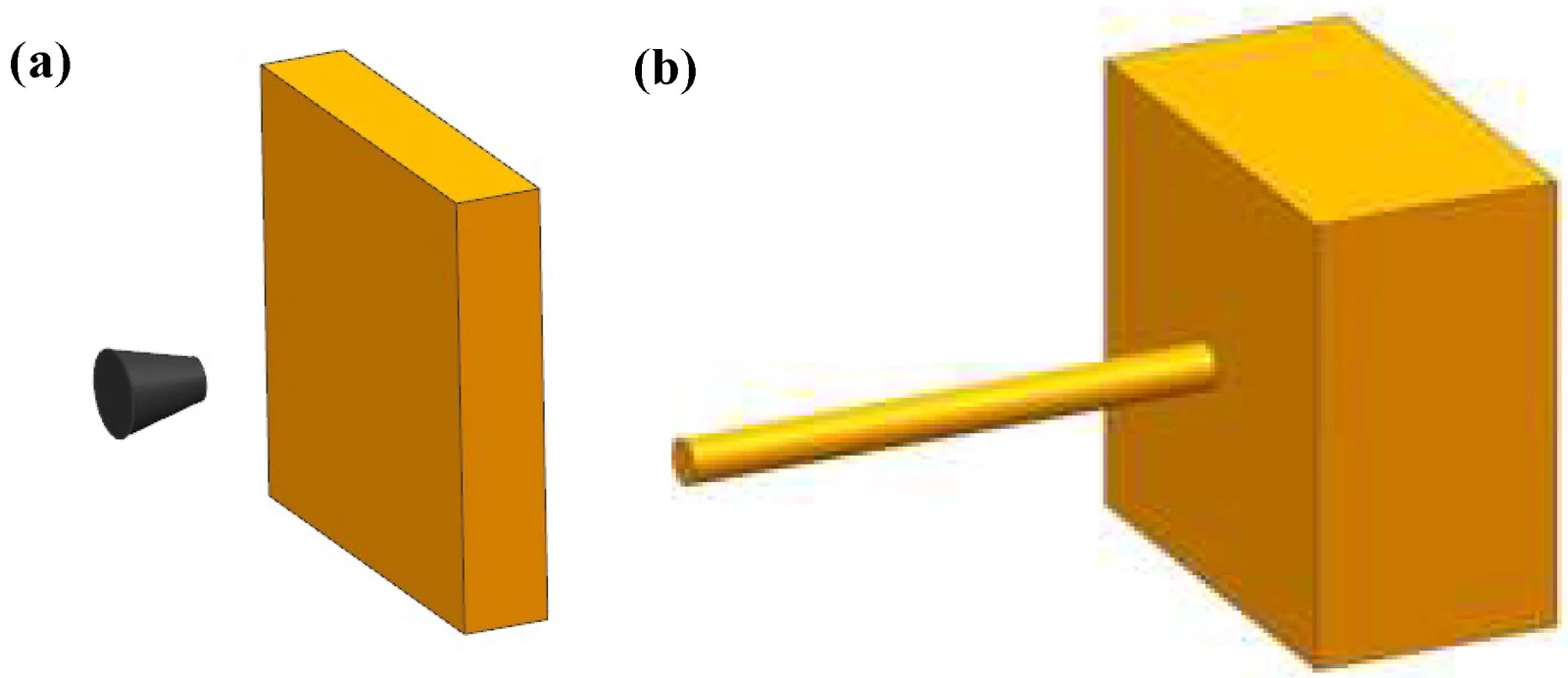
Model geometries of the two typical scenarios. (**a**) 300 g, φ21 mm × 56 mm high-tungsten alloy ogive-nose projectile impacting a 50 mm target; (**b**) 3000 g, φ26 mm × 312 mm (length-to-diameter ratio 12:1) long-rod projectile impacting a 500 mm armor target.

The finite element model consists of a projectile and a target plate. The projectile is modeled as a discrete rigid body, as its deformation during penetration is negligible compared with that of the target material. The target is modeled as a deformable body with elastic-plastic material properties defined through the J-C constitutive model. To identify representative, high-reliability dynamic impact models, two typical scenarios were selected for comparative finite element simulation studies. Two projectile impact simulation schemes with different projectile geometries were designed, and the optimal simulation scenario was determined based on the simulation results. While tungsten projectiles may erode or fragment under ultra-high velocities (>1000 m/s) or repeated hard impacts, such erosion is limited within our studied range (700–1200 m/s) due to the alloy’s high density and yield strength. Moreover, since this study focuses on comparative ranking rather than absolute predictions, any minor erosion would be consistent across all cases and would not affect the relative order of target performance. Figure 1 shows the model geometries of the two typical scenarios:

(1) A 300 g, φ21 mm × 56 mm high-tungsten alloy ogive-nose projectile impacting a 50 mm thick target;
(2) A 3000 g, φ26 mm × 312 mm (length-to-diameter ratio 12:1) long-rod projectile impacting a 500 mm thick target.

#### 2.2.2. Mesh Generation

The target plate is discretized using C3D8R elements—three-dimensional, 8-node linear brick elements with reduced integration. This element type is widely employed in impact simulations due to its computational efficiency and robustness in handling large deformations [30]. The reduced integration formulation, however, is susceptible to hourglass modes, necessitating appropriate hourglass control measures.

A key aspect of the mesh strategy is the local refinement along the projectile penetration path. The target is partitioned into multiple zones, with the central region (the anticipated impact zone) assigned a finer mesh to capture the steep stress and strain gradients associated with penetration. Mesh refinement in this region is achieved through edge seeding along the penetration path.

#### 2.2.3. Hourglass Control

In explicit simulations with reduced-integration C3D8R elements, hourglassing (zero-energy modes) can occur. These spurious checkerboard distortions produce no strain energy yet contaminate stress–strain fields, particularly under large plastic deformation. In impact analyses, hourglassing causes artificial softening and premature failure, so effective control is mandatory for physically meaningful outcomes. To mitigate hourglass instabilities in the reduced-integration C3D8R elements, enhanced hourglass control is activated in Abaqus/Explicit. This formulation provides improved coarse-mesh accuracy and performs better for nonlinear material response at high strain levels compared with the default stiffness formulation. The enhanced hourglass control adds a stabilizing stiffness to the element to suppress spurious zero-energy deformation modes while minimally affecting the physical response.

#### 2.2.4. Boundary Conditions and Contact

The displacements of the four faces parallel to the impact direction on the target are fixed to simulate the clamping or support conditions of an armor plate. The strike face and back face must not be fixed, as simulation verification has confirmed that fixing these faces prevents projectile penetration.

To ensure consistency and robustness in comparative analyses, the projectile is constrained to move only in the impact direction (no lateral displacement or rotation), preventing the unphysical yaw observed in preliminary simulations. This yaw originates from numerical perturbations in the contact algorithm under extreme asymmetric contact conditions. After constraining the projectile to impact-direction motion, the yaw was effectively suppressed, the penetration path returned to normal, and no discernible effect on the projectile velocity–time curve was observed.

The contact interaction between the projectile and the target is defined using global general contact in Abaqus/Explicit. For normal contact behavior, hard contact with separation allowed after contact is prescribed. For tangential contact behavior, the classical Coulomb friction model with shear stress limit is adopted, and a constant friction coefficient of 0.2 is assigned to all contact surfaces between projectile and target, as well as interlayer surfaces of the target.

#### 2.2.5. Analysis Step Settings

The explicit dynamic analysis step requires the determination of an appropriate analysis time length to ensure compatibility between simulation efficiency and resolution of the process evolution. The analysis time is determined based on the impact velocity and target thickness, typically ranging from 0.0002 s to 0.01 s. The theoretical analysis time is estimated as t = h/(0.5 × v), where h is the target thickness and v is the impact velocity. However, due to the nonlinearity of the velocity–time history during penetration, the actual simulation time is adjusted based on the observed penetration process.

### 2.3. Parametric Study Design

A systematic parametric study was conducted to evaluate the influence of key simulation parameters on numerical stability, accuracy, and computational efficiency. Unless otherwise specified, all simulations employed enhanced hourglass control, element deletion, general contact, and conventional boundary conditions. The software version adopted in this work (Abaqus/CAE 2016) does not equip an independent erosion contact release module; instead, the global general contact algorithm automatically identifies and constructs contact pairs on all new exposed surfaces produced by element erosion and deletion. This configuration sustains interfacial contact resistance between the projectile and remaining target material after element failure, eliminating the nonphysical phenomenon that the projectile penetrates deleted elements without resistance and ensuring physically consistent penetration responses.

(1) Material constitutive and damage parameters: Using the small-projectile/thin-target configuration (φ21 mm × 56 mm projectile, 50 mm TC4 target), three parameter sets listed in Table 1 (Sets 1, 2, and 3) were compared. Set 1 adopted literature-reported values with D_4_ = 0.01; Set 2 retained all other parameters while increasing D_4_ to 0.10; Set 3 further adjusted D_2_, D_3_, and D_5_. The same projectile/target geometry, mesh configuration, and boundary conditions were applied across all sets to ensure a fair comparison. The residual velocity, stress contours, and hourglass patterns were analyzed to identify the parameter set that achieves the best balance between numerical stability and physical plausibility. Particular attention was paid to the comparison between D_4_ = 0.01 and D_4_ = 0.10 (Sets 1 and 2), with all other material parameters (D_1_, D_2_, D_3_, D_5_, and the constitutive constants A, B, *n*, C, m) kept constant to isolate the effect of D_4_. The primary evaluation criteria included the presence and severity of hourglass “ghost mesh” distortions in the deformation zone, and the ability to reproduce the complete three-stage penetration process without numerical divergence.
(2) Mesh density and grading strategy: Mesh refinement was applied primarily to the target component. A graded mesh strategy was adopted, with local refinement along the anticipated projectile path and coarser elements in the peripheral regions. Simulations were performed using the large-projectile/thick-target configuration at impact velocities of 500 m/s and 600 m/s. The refined region used an element size of 5.0 mm, while the coarse region used 15 mm. For comparison, a uniformly coarse mesh with 15 mm elements throughout the entire target was also tested. The perforation status, computational time, and hourglass patterns were recorded for each case.
(3) Target dimensions and model scale: For the large-projectile/thick-target configuration, two target sizes were compared: the original dimensions (300 mm × 300 mm × 500 mm) and a reduced size (300 mm × 300 mm × 200 mm). The stress-wave propagation range, the extent of the plastically deformed zone, and the residual velocity deviation (relative to the original case) were monitored to determine whether the reduced model could be adopted without compromising the penetration physics.

Through the above systematic parametric comparisons, an optimal simulation framework—encompassing the material parameter set, mesh strategy, and model configuration—was established and subsequently used for parametric studies on FGTMCs.

### 2.4. Output and Post-Processing

To comprehensively characterize the impact response and verify the numerical stability of the simulations, the following output variables were requested from the Abaqus/Explicit analysis:

(1) Stress and strain fields: Contour plots of von Mises stress (S) and equivalent plastic strain (PEEQ) were saved at intervals of 5–10 μs throughout the penetration process. These plots were used to visualize the evolution of the deformation zone, stress wave propagation, and the formation of adiabatic shear bands. In particular, the PEEQ contours served as the primary indicator for detecting hourglass “ghost mesh” artifacts—uniform or checkerboard-like strain distributions without physical localization were taken as evidence of such artifacts.
(2) Projectile velocity history: A history output was defined for the reference point of the projectile, tracking its velocity component along the impact direction as a function of time. The residual velocity—defined as the projectile velocity after complete perforation or at the moment it exits the target—was extracted from the velocity–time curves and used as a quantitative metric for comparing different material parameter sets and mesh configurations.
(3) Energy quantities: The total kinetic energy (ALLKE), internal energy (ALLIE), and viscous dissipation energy (ALLVD) of the target were monitored throughout the analysis. The energy balance (ALLKE + ALLIE + ALLVD − external work) was checked to ensure global energy conservation, which is a prerequisite for reliable explicit dynamics simulations.
(4) Damage evolution: The scalar damage variable D (ranging from 0 for undamaged material to 1 for completely failed elements) was output for the target elements, allowing the sequence of element deletion and the propagation of the damage path to be tracked.
(5) The scalar damage variable D evolves following displacement-based softening; the characteristic length of each integration point is automatically calculated by Abaqus according to element geometric size, which couples with the 0.01 mm failure displacement to regularize mesh-dependent numerical results.

Post-processing was performed using Abaqus/Viewer 2016 to generate stress and strain contour plots and velocity–time curves for analysis and comparison.

## 3. Results and Discussion

### 3.1. Model Calibration and Parametric Optimization

#### 3.1.1. Sensitivity Analysis of Johnson–Cook Damage Parameter D_4_

**Figure 2:**
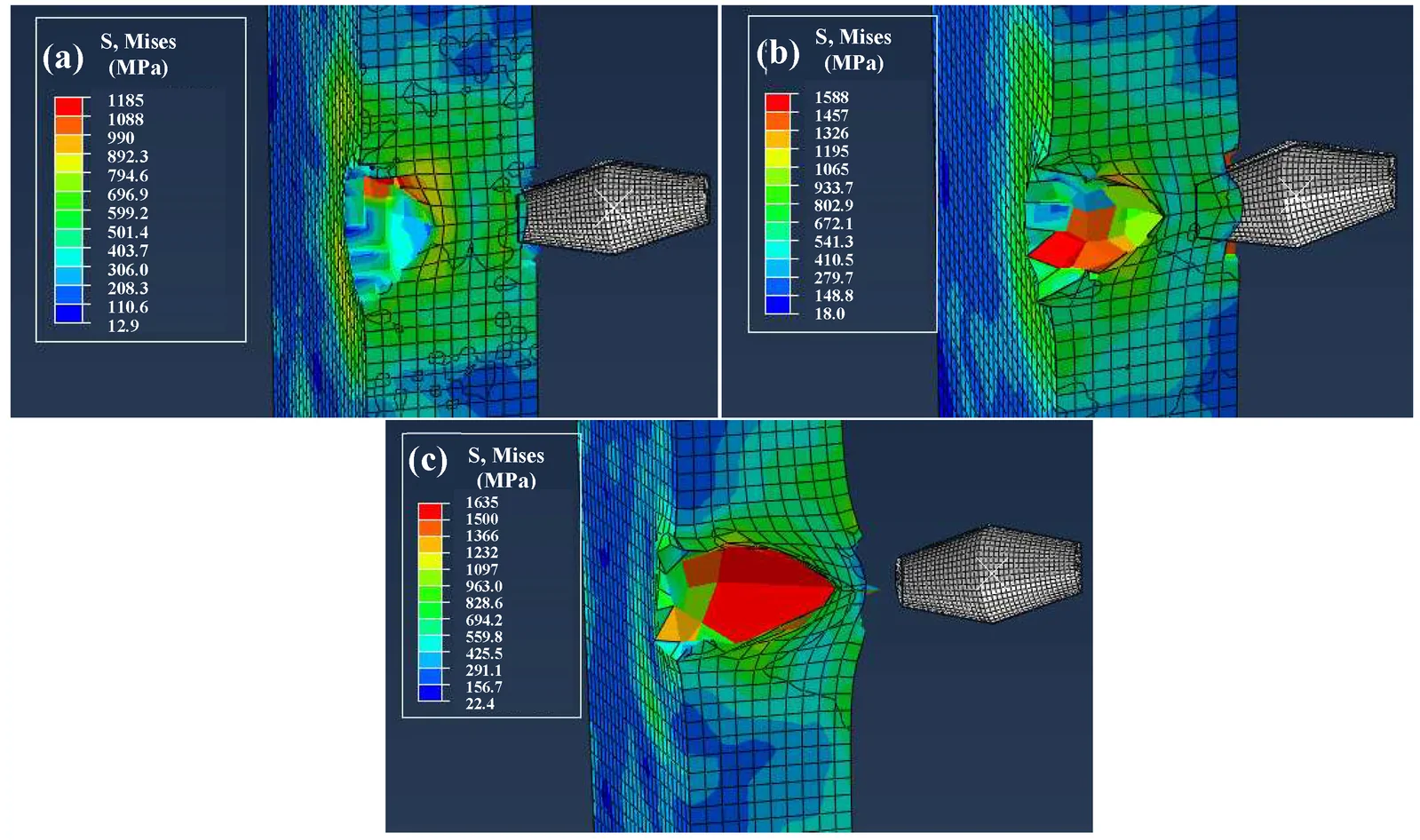
The Mises stress cross-sectional contours for Set 1 (**a**), Set 2 (**b**), and Set 3 (**c**).

Based on the three sets of TC4 titanium alloy parameters (Sets 1, 2, and 3) listed in Table 1, material properties were assigned and numerical simulations of a small projectile impacting a thin target at 1200 m/s were conducted for parametric analysis and optimization. Figure 2 presents the Mises stress cross-sectional contours for Set 1 (Figure 2a), Set 2 (Figure 2b), and Set 3 (Figure 2c). The corresponding residual velocities for the three parameter sets are listed in Table 3. The results show that the projectile completely perforates the target under all three parameter sets; however, the mesh morphologies along the penetration path differ substantially, indicating that the choice of parameter set significantly affects the hourglass “ghost mesh” numerical artifacts.

With Parameter Set 1, severe numerical instability occurs, as shown in Figure 2a. Typical hourglass “ghost mesh” distortion appears in the central deformation zone of the target, caused by unrealistic alternating deformation modes of the elements in this region that accumulate no strain energy, thereby disrupting the stress field and leading to physically implausible penetration predictions. After switching to Parameter Set 2, the hourglass issue is greatly alleviated (Figure 2b), primarily due to the adjustment of the damage parameter D_4_, which will be discussed in detail below.

When D_4_ = 0.01, the fracture strain exhibits low sensitivity to strain rate. High-strain elements undergoing large deformation cannot effectively dissipate energy, readily exciting zero-energy deformation modes in the reduced-integration elements. At this setting, neither mesh refinement nor adjustment of hourglass control parameters can eliminate the persistent hourglass instability in the central deformation zone. Increasing D_4_ to 0.1 markedly improves numerical stability, enabling the simulation to reproduce the three typical penetration stages: cratering, severe plastic deformation, and complete perforation.

Compared with Parameter Set 2, Parameter Set 3 increases D_2_ and D_3_ while reducing D_5_ to 0. As seen in Figure 2c, the numerical artifacts are further suppressed. The J-C damage parameters D_2_ and D_3_ control the amplification of fracture strain by plastic strain and triaxial stress; larger values raise the critical fracture strain and delay element deletion. D_5_ represents the thermal-softening effect on fracture; setting it to zero means that adiabatic heating no longer lowers the fracture threshold. Consequently, with Parameter Set 3, elements undergo larger plastic deformation before failure, preventing premature distortion and spurious strain-energy accumulation, thus suppressing the hourglass zero-energy modes at their source. As a result, “ghost mesh” artifacts are significantly reduced.

From Table 3, the residual velocities for Sets 1, 2, and 3 are 1015, 920, and 825 m/s, respectively, which are consistent with the observations in Figure 2a–c and the corresponding analysis. Under high-strain-rate impact, the strain-rate sensitivity of fracture in TC4 titanium alloy should be set to a relatively high value so that excessively distorted elements are promptly deleted, preventing severely deformed elements from persisting in the mesh. This is a core optimization measure for achieving numerical stability in our simulation framework. In summary, the use of Parameter Set 3 for material property definition yields relatively reliable simulation results. 

#### 3.1.2. Mesh Sensitivity and Convergence

**Figure 3:**
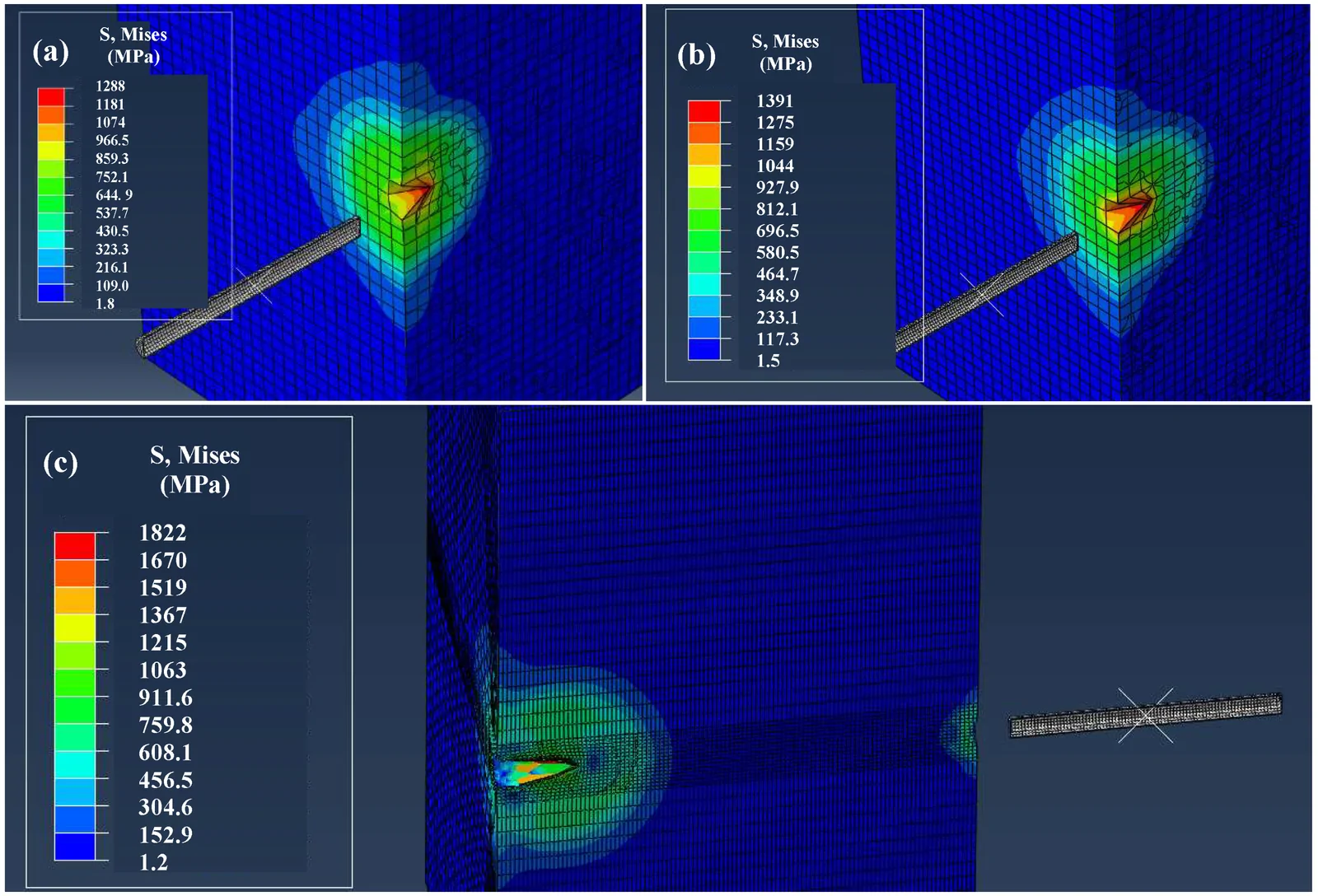
Comparison of penetration contours with different mesh sizes. (**a**) 500 m/s, coarse mesh: penetration depth noticeably too shallow, blurred shear band contours, large calculation errors; (**b**) 500 m/s, fine mesh (threefold refinement): improved calculation accuracy and data precision, but computation time increased by 3–5 times with diminishing returns; (**c**) 600 m/s, coarse mesh: penetration depth noticeably too shallow, blurred shear band contours, large calculation errors.

The influence of mesh size on impact simulation accuracy and computational efficiency is shown in Figure 3. Coarse meshes were computationally efficient but produced penetration depth predictions that were noticeably too shallow and failed to resolve the boundaries of the plastic penetration zone. In the large projectile/thick target configuration, contradictory results were observed: the coarse mesh predicted no perforation at 500 m/s (Figure 3a), while the refined mesh predicted complete perforation at the same velocity (Figure 3b), indicating that the coarse mesh significantly underestimated the local strain concentration effects. At 600 m/s, the coarse mesh model still failed to predict perforation, while the refined mesh model (Figure 3c) predicted perforation, further highlighting the substantial influence of mesh size on simulation accuracy.

Mesh size in our continuum-based simulations does not represent actual grain size or dislocation substructures. Instead, the Johnson–Cook constitutive model and damage criterion implicitly incorporate microstructural effects through calibrated parameters (e.g., hardening exponent, rate sensitivity). A finer mesh resolves steep strain gradients and localizes deformation within shear bands, which is essential for accurate penetration depth and failure predictions. Conversely, an overly coarse mesh smears these gradients, underestimates plastic strain concentration, and delays element deletion, resulting in shallower penetration or false non-perforation. Thus, the improvement with refinement is purely a numerical convergence issue—ensuring the FE solution approaches the continuum solution for the given constitutive law, not a direct simulation of microstructural features. 

Based on comprehensive evaluation across all conditions, a graded mesh with local refinement along the penetration path was identified as the optimal strategy: the mesh size in the central penetration path region of the target was refined to capture severe strain gradients, while coarser meshes were used in other regions. This strategy achieves an optimal balance between accuracy and efficiency, maintaining engineering precision while keeping computation time within acceptable limits.

#### 3.1.3. Hourglass Element Formation and Mitigation

The most persistent numerical challenge encountered in this study was the formation of hourglass elements (also referred to as “ghost mesh” or zero-energy deformation modes) in the central deformation region of the target, particularly under high impact velocities and thick target conditions, as shown in Figure 2a and Figure 3. The elements in the penetration path of the central region exhibited the following characteristics:

(1) Unrealistic deformation patterns (hourglassing) without associated strain energy;
(2) Occurrence primarily in regions along the penetration path where elements should have undergone high deformation and deletion, but instead remained undeformed and undeleted;
(3) Resistance to elimination through material parameter adjustment alone;
(4) Severe compromise of stress and strain predictions in the affected regions.

Hourglass elements were particularly pronounced in the large projectile/thick target configuration, where the extensive deformation zone and long simulation duration exacerbated numerical instabilities. Even with enhanced hourglass control activated, complete elimination of hourglass modes proved challenging in this configuration.

For the small projectile/thin target configuration (φ21 mm × 56 mm projectile, 50 mm TC4 target), hourglass elements, though observed, could be effectively controlled through a combination of measures:

(1) Enhanced hourglass control: Activation of the enhanced hourglass control formulation in Abaqus/Explicit;
(2) Optimization of D_4_ and damage evolution displacement parameters: Setting D_4_ = 0.1 and damage evolution displacement = 0.01 significantly improved numerical stability;
(3) Appropriate mesh refinement: Sufficient mesh density in the deformation zone without over-refinement.

For the large projectile/thick target scenario, the above three hourglass control measures showed limited improvement in the present simulation verification compared with the small-target case. This observation is consistent with the known behavior of reduced-integration elements under extreme deformation conditions. As noted in the Abaqus/Explicit documentation, while the enhanced hourglass control formulation provides improved coarse-mesh accuracy and performs better for nonlinear material response at high strain levels compared with the default stiffness formulation, its effectiveness can be compromised when elements undergo prolonged and severe distortion [31]. Similar numerical challenges associated with hourglass modes in reduced-integration elements subjected to large deformations have been reported in the literature [32]. This suggests that for extremely thick targets, additional measures beyond the standard hourglass controls—such as further mesh refinement, alternative element formulations (e.g., fully integrated elements), or combined hourglass control schemes—may be required, although a systematic parametric investigation of this aspect remains to be explored in future work.

**Figure 4:**
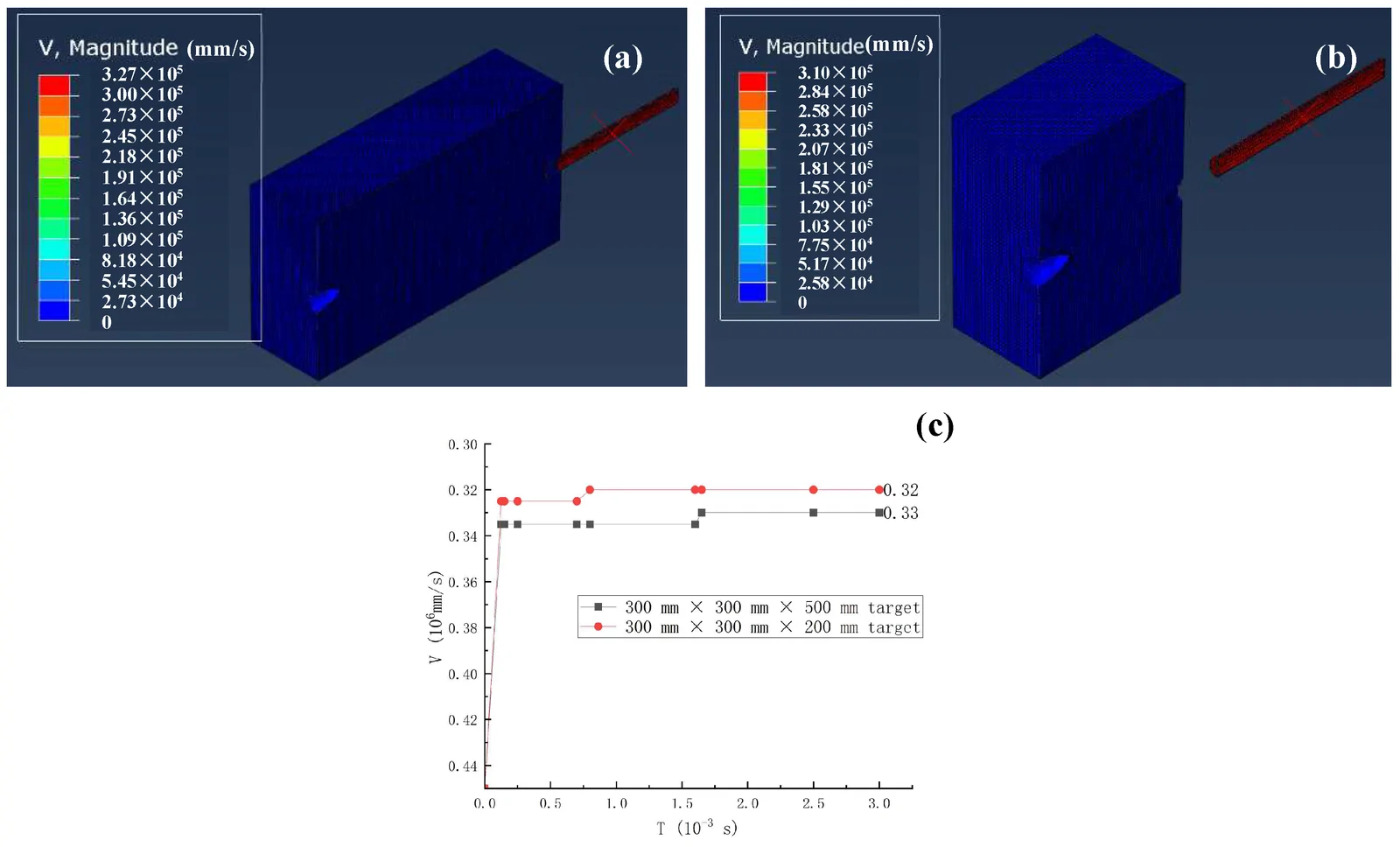
Simulation of 450 m/s projectile perforation of TC4 in the large projectile/thick target configuration: (**a**) velocity field contour for the 300 mm × 300 mm × 500 mm target, (**b**) velocity field contour for the 300 mm × 300 mm × 200 mm targe, (**c**) comparison of velocity evolution.

Reducing the target thickness from 500 mm to 200 mm effectively removed regions prone to hourglass formation without affecting the penetration dynamics, as shown in Figure 4. Removing the “ghost regions” not reached by stress waves or deformation had minimal impact on the simulation results; the effect on projectile deceleration was negligible (residual velocity deviation < 4%), but computation time was reduced by approximately 70%. However, reducing the thickness of the target for simulation purposes lost the original significance of thick armor simulation. Furthermore, the velocity–time curve trends (a rapid deceleration at the strike face followed by a prolonged period of constant velocity before deceleration at the back face) indicated that the “ghost mesh” problem remained prominent. Through comparison, the small projectile/thin target model demonstrated significant superiority over the large projectile/thick target model in terms of numerical stability, computational efficiency, and clarity of mechanism analysis, making it more suitable for parametric analysis and material response mechanism studies. Therefore, for simulations of titanium alloys, TMCs, and FGTMCs, the small projectile/thin target configuration (φ21 mm × 56 mm projectile, 50 mm TC4 target) is the superior model. The large projectile/thick target model is recommended only for final engineering validation after the parameter system has been fully optimized. The 700 m/s small-caliber tungsten projectile impacting a 50 mm TC4 plate can be selected as the standard simulation condition, with other homogeneous materials or graded materials then substituted for simulation, evaluation, and comparison.

The small-projectile/thin-target setup is recommended primarily for parametric studies and mechanistic analyses, where numerical stability, computational efficiency, and clear visualization of the three-stage damage evolution are paramount. For direct engineering predictions—such as drone armor or aerospace panels—impact conditions (caliber, velocity, thickness, constraints) may differ substantially, making the large-projectile/thick-target configuration more representative despite its numerical difficulties. We thus suggest using the small-model approach for rapid screening and optimization of material/gradient designs, while final validation against full-scale experiments or full-geometry simulations remains essential for practical defense applications.

### 3.2. Material System and Gradient Model Simulation Validation

#### 3.2.1. Property Assignment for TC4 and Typical Titanium Matrix Composites

Based on the calibrated J-C constitutive and damage model parameters and experimental performance testing, this study further designed a material system encompassing TC4 and three typical titanium matrix composites. Table 4 presents a comparison of key J-C constitutive parameters for the four homogeneous materials. The TiC_p_ content and size vary across the different material systems, with their constitutive parameters established based on the rule of mixtures and reported mechanical property data for composites from the literature.

#### 3.2.2. Material System Parameterization and Gradient Model Development

For simulation of FGTMCs, model construction must account for the material bonding configuration and the characteristics of material property variation. Two technical routes are available in Abaqus for implementing continuous smooth gradients: (1) the USDFLD subroutine, which assigns field variables as continuous functions of coordinates for element-scale gradation; and (2) the layered partition method, which divides the target into multiple layers with progressively varying properties and shared nodes for displacement continuity—no macroscopic interfaces are introduced. We adopt the layered partition method in this study. The stepwise material variation across the layers effectively captures the metallurgical bonding, interface-free transition, and smooth property gradation characteristic of titanium-based gradient composites. With sufficient layer divisions, the property profile closely matches that of a continuously graded model, accurately reproducing the “hard outer, tough inner” gradient response and satisfying the comparative simulation requirements of this work.

**Figure 5:**
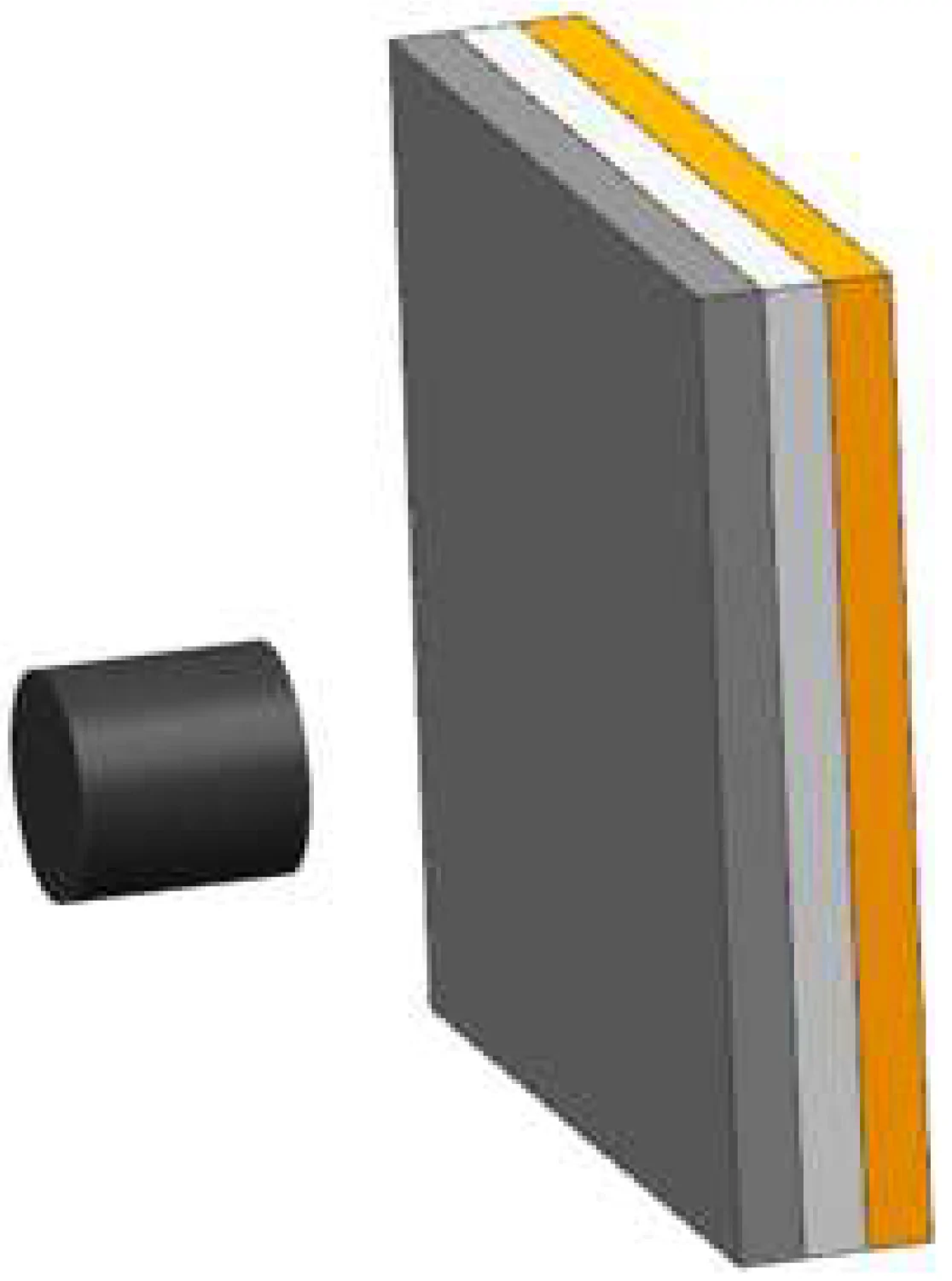
Schematic of projectile penetration into the target.

Using a three-layer graded material as an example for process optimization, Figure 5 presents a schematic of projectile penetration into the target, where the target consists of three layers with distinct material properties. The three material constituents of the graded material are assigned separately after partitioning, which better captures the same-matrix transition and metallurgical bonding characteristics of different layers in the graded material. The core strategies for establishing the FGTMC finite element model are as follows:

(1) The target is divided along the thickness direction into multiple independent regions, with each region assigned different material properties corresponding to different layers;
(2) Adjacent layers are connected through shared nodes to enforce displacement continuity, simulating metallurgical bonding at the interfaces;
(3) No macroscopic interface is defined between graded transition layers; instead, a smooth performance transition is achieved through gradual variation in element properties.

The advantage of this modeling approach lies in its flexibility to adjust layer thicknesses and material combinations without altering the mesh topology, providing a convenient simulation platform for subsequent parametric optimization of graded structures.

### 3.3. Penetration Process Analysis

#### 3.3.1. Three-Stage Penetration Mechanism

**Figure 6:**
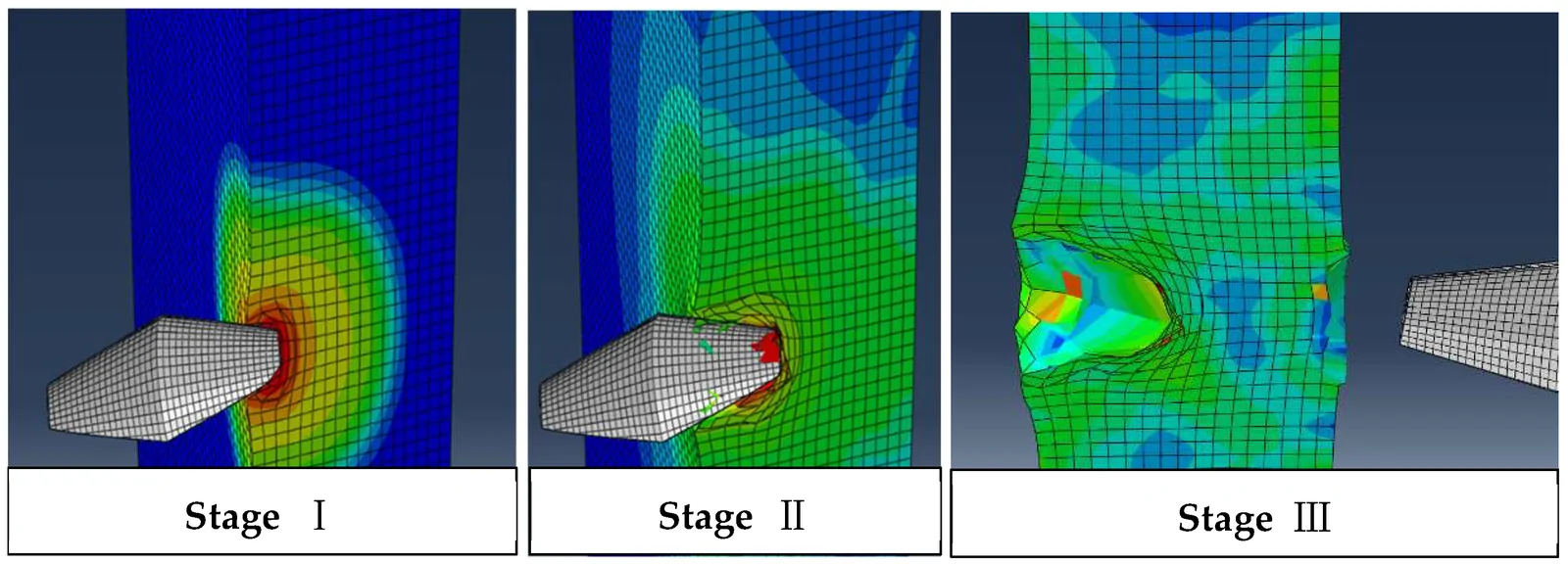
Schematic of the three-stage projectile penetration damage mechanism of armor plates. Stage I—Cratering: high-speed projectile produces ultra-high local pressure on the target surface, plastic cutting forms an impact crater; requires high-hardness strike face to blunt the projectile tip. Stage II—Penetration: projectile continuously extrudes the matrix to form adiabatic shear bands, stress waves diffuse to the back face; the transition layer needs matching strength and toughness to absorb plastic deformation energy. Stage III—Spallation/perforation: reflected stress waves cause back plate fragmentation; high-toughness back layer prevents secondary fragment damage. Note: The colors in the figure transition from red to blue, corresponding to a gradual decrease in stress.

Figure 6 shows the simulation of a 1200 m/s small projectile penetrating the TMCs1/TMCs2/TC4 graded layer. The simulation results successfully captured the three characteristic stages of penetration in the FGTMC target subjected to high-velocity projectile impact, reproducing the cratering, plastic penetration, and back-face spallation three-stage damage evolution of titanium alloys under impact, consistent with classical penetration mechanics [33]. This provides reliable numerical support for the structural optimization of graded armor materials. The visualized results are presented as follows:

Stage I—Cratering: Upon initial impact, the high-velocity projectile strikes the target surface. The hard tungsten alloy projectile (HV 500–700) “hard-penetrates” the strike face. This stage is characterized by localized high pressure in the shallow surface layer, causing severe plastic deformation and cutting to form a crater. The simulation results demonstrate that this stage requires high hardness at the strike face to blunt the projectile nose and high strength to resist plastic deformation.

Stage II—Penetration: As the projectile continues to advance, it exerts sustained compression on the armor material, generating adiabatic shear bands. The surrounding armor material undergoes plastic deformation and flow, while stress waves propagate outward and toward the back face. This stage requires the intermediate layer to possess both high strength to resist impact and high toughness to absorb energy through deformation. The transition layer between the hard strike face and the tough back face serves to support the hard surface layer and prevent interlayer stress concentration.

Stage III—Perforation/Spallation: In the final stage, the projectile either completely penetrates the target or stress waves cause discing or spallation on the back face. The resulting fragments can cause secondary damage to personnel or equipment. This stage requires high toughness at the back face to absorb energy through plastic deformation and maintain structural integrity, preventing fragmentation.

#### 3.3.2. Velocity Threshold and Penetration Depth

**Figure 7:**
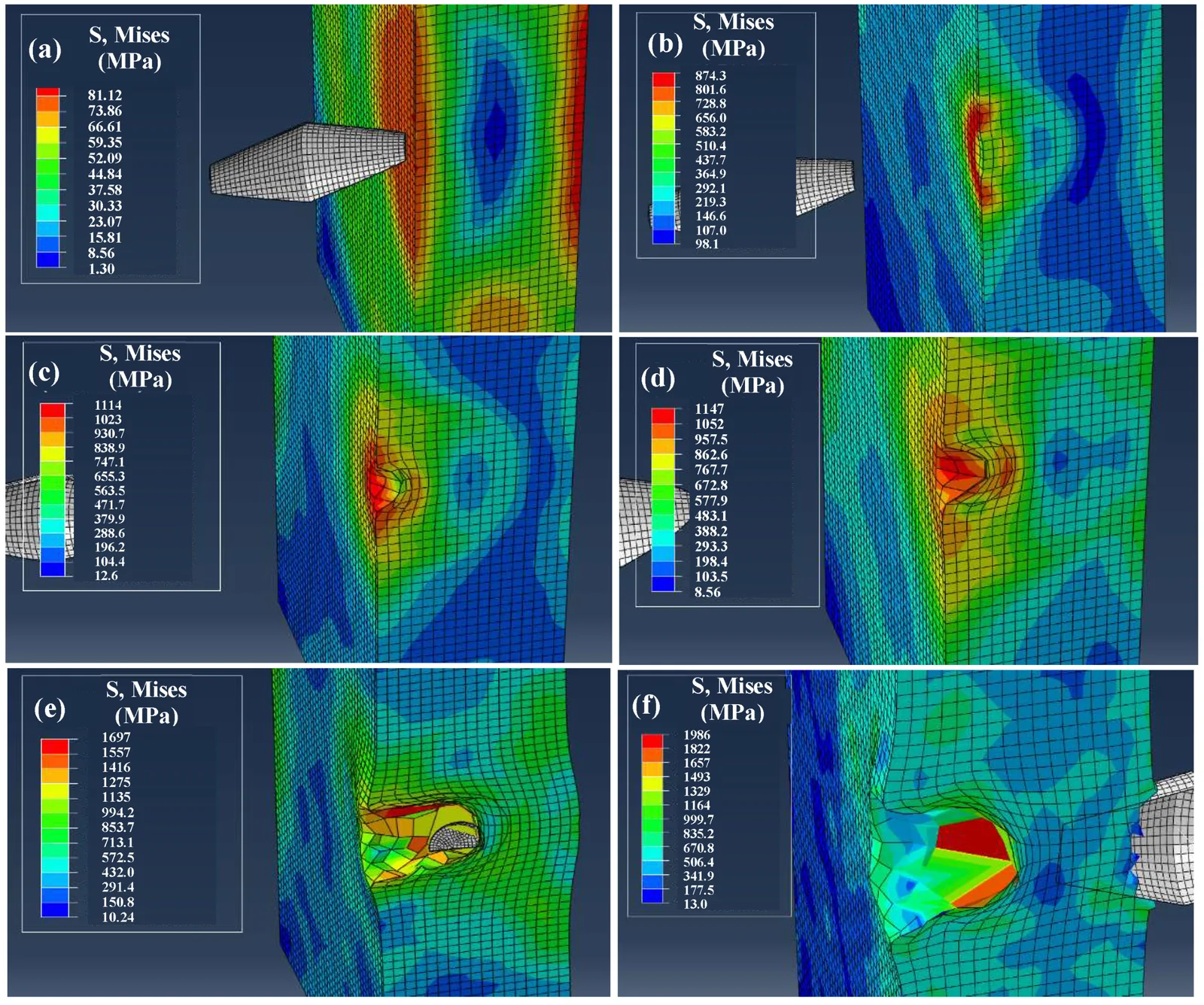
Dynamic impact simulations of the small projectile/thin target scenario penetrating FGTMCs at different impact velocities: (**a**) 20 m/s, (**b**) 80 m/s, (**c**) 200 m/s, (**d**) 300 m/s, (**e**) 700 m/s, (**f**) 1200 m/s.

In the small projectile/thin target scenario, systematic dynamic impact simulations were conducted over an impact velocity range of 20–1200 m/s. The penetration depth contours at different velocities, shown in Figure 7, reveal a clear perforation velocity threshold. Table 5 summarizes the impact responses of the small-projectile/thin-target configuration (FGTMC target) at different velocities. At a low impact velocity of 20 m/s, the target undergoes minimal deformation, with the response confined to elastic deformation in the surface layer. When the velocity increases to 80 m/s, plastic deformation begins in the surface layer, marking the initiation of the cratering stage (Stage I). At 200 m/s, the characteristics of the penetration stage (Stage II) begin to appear, with the projectile’s extrusion effect on the target gradually intensifying. At 300 m/s, Stage II is fully developed, with adiabatic shear bands extending inward along the penetration path. When the velocity reaches 700 m/s, the perforation stage (Stage III) is initiated, with a significant increase in penetration depth; however, the back face of the target remains in the plastic deformation stage without spallation, and no hourglass element distortion occurs throughout the calculation process. When the velocity is further increased to 1200 m/s, the projectile completely perforates the target, resulting in overall structural failure. Considering numerical stability, richness of physical phenomena, and computational economy across all velocity conditions, 700 m/s is identified as the optimal simulation condition—at this velocity, the projectile does not fully perforate the target, a relatively deep penetration crater is formed, the back face remains in the deformation stage without spallation, and complete data for the three-stage cratering–penetration–perforation evolution are provided, while avoiding the numerical complications associated with complete perforation or extensive fragmentation.

#### 3.3.3. Design Criteria for Impact-Resistant FGTMCs

Based on our simulation results, several key design criteria for impact-resistant FGTMCs can be established. The strike face should possess high hardness and yield strength—achieved through a higher TiC particle fraction—to effectively blunt the projectile nose and minimize crater depth. The intermediate layers should be designed with a gradual reduction in hardness accompanied by an increase in ductility, which helps alleviate interfacial stress concentrations and dissipate kinetic energy through plastic flow. The back face requires superior fracture toughness and ductility to absorb residual energy and prevent catastrophic spallation. In addition, the thickness ratios between layers and the steepness of the property gradient are critical factors: a smooth gradient minimizes internal stress wave reflections, whereas a sharp gradient tends to promote interfacial delamination. The validated simulation framework established in this study enables rapid evaluation of these design parameters and provides practical guidelines for tailoring FGTMC architectures to meet specific impact-velocity and protection-level requirements.

## 4. Conclusions

This study presents a systematic investigation of the dynamic impact simulation methodology for FGTMCs using Abaqus/Explicit, leading to the following conclusions:

(1) A standardized simulation parameter system for dynamic impact of TC4 titanium alloy has been established. Through systematic parametric analysis, the optimized Johnson–Cook damage parameter D_4_ = 0.1 effectively suppresses hourglass “ghost mesh” numerical distortion in the central deformation zone under high strain rates, significantly improving computational stability. A graded mesh with 1.5–2 mm refinement along the penetration path achieves a balance between the accuracy of capturing stress–strain gradients and computational efficiency. The small-caliber tungsten projectile (φ21 mm × 56 mm, 300 g) impacting a 50 mm TC4 target plate is identified as the optimal projectile–target configuration for mechanism studies, with numerical stability and computational economy significantly superior to the large projectile/thick target scheme.
(2) A dynamic impact simulation model encompassing both homogeneous and graded material systems has been constructed. A material property parameter system covering TC4 and three typical TMCs has been designed, and a finite element model for functionally graded titanium matrix composites (FGTMCs) has been established through a multi-zone section definition method, enabling the graded assignment of material properties along the thickness direction. This provides an extensible simulation platform for multi-parameter optimization of graded armor structures.
(3) The three-stage penetration damage evolution law has been reproduced and the impact resistance mechanism of the graded structure has been elucidated. The simulation results clearly present the three-stage damage evolution process—cratering (requiring high hardness at the strike face), penetration (requiring matching strength and toughness in the transition layer), and spallation (requiring high toughness at the back face)—validating the physical matching relationship between the “hard outer, tough inner” gradient configuration and the multi-stage penetration load characteristics of projectiles. The established simulation methodology can effectively guide the optimization design of key structural parameters for graded armor materials, providing reliable numerical support for the development of lightweight, high-impact-resistance graded composites.

## Figures and Tables

**Table 1:** Preliminary Johnson–Cook constitutive model parameters for TC4 titanium alloy.

Parameter	Set 1 [26]	Set 2 [27]	Set 3 [28]	Unit	Physical Meaning
ρ	4430	4430	4430	kg/m^3^	Material density
E	110	110	110	GPa	Elastic modulus
ν	0.33	0.33	0.33	—	Poisson’s ratio
A	750	750	750	MPa	Static yield strength
B	500	500	500	MPa	Strain hardening coefficient
*n*	0.5	0.5	0.5	—	Hardening exponent
C	0.012	0.012	0.012	—	Strain rate sensitivity factor
m	1.1	1.1	1.1	—	Thermal softening exponent
D_1_	0.1	0.1	0.1	—	Damage constant 1
D_2_	0.270	0.270	0.8	—	Damage constant 2
D_3_	0.48	0.48	1.5	—	Damage constant 3
D_4_	0.01	0.1	0.1	—	Strain rate damage sensitivity
D_5_	3.87	3.87	0	—	Temperature damage sensitivity
Tr	293	293	293	K	Reference temperature
Tm	1933	1933	1933	K	Melting temperature
*ε* ^*0^	0.001	0.001	0.001	s^−1^	Reference strain rate
Damage displacement	0.01	0.01	0.01	—	

**Table 2:** Impact velocities in protective scenarios.

Category	Impact Velocity (m/s)	Remarks
Armor-piercing projectile	500–1800	Metallic armor, composite armor
Shaped charge jet	1000–3000	Metal jet drilling; often requires reactive armor; complex process
SHPB test	4–35	Medium-to-high strain rate selected for titanium alloy tests (20 m/s)
Flat-nosed projectile	160–320	Wide applicability

**Table 3:** Residual velocities corresponding to Parameter Sets 1–3.

Parameter Set	Residual Velocity (m/s)
Set 1	1015
Set 2	920
Set 3	825

**Table 4:** Comparison of key J-C constitutive parameters for the four homogeneous materials.

Parameter	TC4	TMCs1	TMCs2	TMCs3	
ρ	4510	4510	4510	4510	kg/m^3^
E	110	165	134.5	143	GPa
ν	0.33	0.33	0.33	0.33	—
A	750	1125	937.5	975	MPa
B	500	600	550	575	MPa
*n*	0.5	0.45	0.55	0.48	—
C	0.012	0.012	0.012	0.012	—
m	1.1	1.1	1.1	1.1	—
D_1_	0.1	0.07	0.12	0.85	—
D_2_	0.8	0.8	0.8	0.8	—
D_3_	1.5	1.5	1.5	1.5	—
D_4_	0.1	0.1	0.1	0.1	—
D_5_	0	0	0	0	—
Tr	293	293	293	293	K
Tm	1933	1933	1933	1933	K
*ε* ^*0^	0.001	0.001	0.001	0.001	s^−1^
Damage displacement	0.01	0.01	0.01	0.01	

**Table 5:** Summary of impact response at different velocities for the small-projectile/thin-target configuration (FGTMC target).

Impact Velocity (m/s)	Penetration Stage Observed	Key Features
20	Elastic rebound	Negligible plastic deformation; target remains intact.
80	Incipient cratering (Stage I)	Localized plastic deformation at strike face; shallow crater begins.
200	Early penetration (Stage II)	Projectile extrusion intensifies; adiabatic shear bands start to form.
300	Fully developed penetration (Stage II)	Clear shear bands; plastic flow extends inward; no rear-face damage.
700	Pre-perforation (Stage II–III transition)	Deep crater, significant plastic penetration; back face deformed but not spalled; no hourglass artifacts.
1200	Complete perforation (Stage III)	Target perforated; rear-face spallation and fragmentation.

## Data Availability

The original contributions presented in this study are included in the article. Further inquiries can be directed to the corresponding author.

## References

[1] Boyer R.R. (2010). Attributes, characteristics, and applications of titanium and its alloys. JOM.

[2] Hayat D.M., Singh H., He Z., Cao P. (2019). Titanium metal matrix composites: An overview. Compos. Part A.

[3] Zhang W., Xu J. (2022). Advanced lightweight materials for automobiles: A review. Mater. Des..

[4] Hao F., Xin S.W., Mao Y.C. (2020). Overview of the application of titanium alloy in armor field. Mater. Rep..

[5] Zhang J.B. (2023). Exploration of titanium alloy technology for armor of new generation ground combat platforms. Tank. Armoured Veh..

[6] Wang J.X., Yang J.J., Liu K. (2023). Study on the damage characteristics of metal sandwich panels under the combined action of shock waves and randomly distributed fragments. Shipbuild. China.

[7] Gupta N., Basu B., Prasad B.V., Vemuri M. (2012). Ballistic studies on TiB2-Ti functionally graded armor ceramics. Def. Sci. J..

[8] Shojaei P., Trabia M., O’Toole B., Jennings R., Zhang X., Liao Y. (2020). Enhancing hypervelocity impact resistance of titanium substrate using Ti/SiC metal matrix nanocomposite coating. Compos. Part B.

[9] Aman G., Rajesh K., Jaiveer S. (2022). A review of microstructure and texture evolution during plastic deformation and heat treatment of β-Ti alloys. J. Alloys Compos..

[10] Aman G., Kisni R.K., Amit K., Parihar M.S. (2018). Investigations on the effect of heating temperature and cooling rate on evolution of microstructure in an α + β titanium alloy. J. Mater. Res..

[11] Diksha M., Khatirkar R.K., Gupta S.K., Gupta A. (2022). Microstructure evolution and corrosion behaviour of a high Mo containing α + β titanium alloy for biomedical applications. J. Alloys Compos..

[12] Dong L.L., Wang Y.M., Cui W.F., Sun G.D., Xu J.J., Li M.J., Zhou L., Zhang Y.S. (2023). Research progress on interface structure and performance optimization of nano carbon reinforced titanium based composites. Prog. Mater. China.

[13] Zhang C., Kong F., Xiao S., Zhao E., Xu L., Chen Y. (2012). Evolution of microstructure and tensile properties of in situ titanium matrix composites with volume fraction of (TiB+TiC) reinforcements. Mater. Sci. Eng. A.

[14] Huang L.J., Geng L., Peng H.X. (2015). Microstructurally inhomogeneous composites: Is a homogeneous reinforcement distribution optimal?. Prog. Mater. Sci..

[15] Shao X., Wang W., Xie J., Qin W.D., Wang A.Q., Ma D.Q., Mao Z.P., Liu P., Guo Q.Y. (2026). Construction, microstructure, and properties of Ti6Al4V/TiC functionally graded material with porous surface. Acta Metall. Sin..

[16] Borvik T., Langseth M., Hopperstad O., Malo K. (2002). Perforation of 12mm thick steel plates by 20mm diameter projectiles with flat, hemispherical and conical noses. Int. J. Impact Eng..

[17] Hazell P.J., Fellows N.A., Hetherington J.G. (1998). A note on the behind armour effects from perforated alumina/aluminium targets. Int. J. Impact Eng..

[18] Meyers M.A. (1994). Dynamic Behavior of Materials.

[19] Zhang C., Cao J.W., Guo J.B., Liu F.F., Wang C., Hasi G.W., Guo Z.Z. (2020). The research on transparent armor material technology. J. Phys. Conf. Ser..

[20] Jeje S.O., Marazani T., Shongwe M.B. (2024). Impact behavior of spark plasma sintered Ti–Al–Mo/TiN composites: A finite element analysis approach using Abaqus CAE. Beni-Suef Univ. J. Basic Appl. Sci..

[21] Dominik G., Wojciech M., Michał M., Głowacka A., Kraśkiewicz C. (2021). Energy Absorbing Properties Analysis of Layers Structure of Titanium Alloy Ti6Al4V during Dynamic Impact Loading Tests. Materials.

[22] Bobbili R., Ramakrishna B., Madhu V. (2017). Dynamic compressive behavior and fracture modeling of Titanium alloy IMI 834. J. Alloys Compd..

[23] Gama B.A., Lopatnikov S.L., Gillespie J.W. (2004). Hopkinson bar experimental technique: A critical review. Appl. Mech. Rev..

[24] Johnson G.R., Cook W.H. A constitutive model and data for metals subjected to large strains, high strain rates and high temperatures. Proceedings of the 7th International Symposium on Ballistics.

[25] Johnson G.R., Cook W.H. (1985). Fracture characteristics of three metals subjected to various strains, strain rates, temperatures and pressures. Eng. Fract. Mech..

[26] Xue J., Wang J., Xiong R., Dong W., Zhang H., Liu J., Geng X. (2025). A study of Johnson-Cook modeling under transient shocks. J. Phys. Conf. Ser..

[27] Dong K., Lv W., Sun Y., Wang P., Tian H., Zhou F., Du Z., Du C. (2025). Research on the dynamic mechanical properties of selective laser melting TC4 titanium alloy and the construction of J-C model. Mater. Today Commun..

[28] Yin W., Liu Y., He X., Tian Z. (2024). Parametric Analysis and Improvement of the Johnson-Cook Model for a TC4 Titanium Alloy. Metals.

[29] Jia L., Li C., Zou X., Yao X.H. (2020). Deformation and destruction of TC4 titanium alloy plate under the bird impact. Chin. J. High. Press. Phys..

[30] Shao X., Wang W., Xie J., Ma D., Wang J., Mao Z. (2026). Homogeneous, laminated, and functionally graded TiC_p_/TC4 composites: Microstructure, mechanical properties, and dynamic impact behavior. J. Mater. Res. Technol..

[31] Dassault Systèmes Simulia Corp (2016). ABAQUS Analysis User’s Manual, Version 2016.

[32] Reese S. (2007). A large deformation solid-shell concept based on reduced integration with hourglass stabilization. Int. J. Numer. Methods Eng..

[33] Banerjee D., Williams J.C. (2013). Perspectives on titanium science and technology. Acta Mater..

